# Getting lipids from glycerol: new perspectives on biotechnological exploitation of *Candida freyschussii*

**DOI:** 10.1186/1475-2859-13-83

**Published:** 2014-06-07

**Authors:** Stefano Raimondi, Maddalena Rossi, Alan Leonardi, Michele Maria Bianchi, Teresa Rinaldi, Alberto Amaretti

**Affiliations:** 1 Department of Life Sciences, University of Modena and Reggio Emilia, via Campi 183, 41100 Modena, Italy; 2 Department of Biology and Biotechnology Charles Darwin, University of Rome ‘La Sapienza’, Piazza Aldo Moro 5, 00185 Rome, Italy

## Abstract

**Background:**

Microbial lipids represent a valuable alternative feedstock for biodiesel production when oleaginous microbes are cultured with inexpensive substrates in processes exhibiting high yield and productivity. In this perspective, crude glycerol is among the most promising raw materials for lipid production, because it is the costless residual of biodiesel production. Thus, cultivation of oleaginous yeasts in glycerol-based media is attracting great interest and natural biodiversity is increasingly explored to identify novel oleaginous species recycling this carbon source for growth and lipid production.

**Results:**

Thirty-three yeasts strains belonging to 19 species were screened for the ability to grow and produce intracellular lipids in a pure glycerol-based medium with high C/N ratio. A minority of them consumed most of the glycerol and generated visible lipid bodies. Among them, *Candida freyschussii* ATCC 18737 was selected, because it exhibited the highest lipid production and glycerol conversion yield. Lipid production in this strain was positively affected by the increase of C/N ratio, but growth was inhibited by glycerol concentration higher than 40 g/L. In batch cultures, the highest lipid production (4.6 g/L), lipid content of biomass (33% w/w), and lipid volumetric productivity (0.15 g/L/h) were obtained with 40 g/L glycerol, during the course of a 30-h process. Fed-batch cultivation succeeded in preventing substrate inhibition and in achieving a high cell-density culture. The improved lipid production and volumetric productivity reached the remarkable high level of 28 g/L and 0.28 g/L/h, respectively. The lipids accumulated by *C. freyschussii* ATCC 18737 have similar fatty acid composition of plant oil indicating their potential use as biodiesel feedstock. Calculated physicochemical properties of a biodiesel produced with the lipids from *C. freyschussii* ATCC 18737 are expected to meet the European and American standards, being equal to those of rapeseed and palm biodiesel.

**Conclusions:**

*C. freyschussii* ATCC 18737 could be considered an interesting microorganism for utilization in biofuel industry. Cultivation of this yeast in media containing crude glycerol should be investigated deeper in order to evaluate whether it may find application in the valorization of the waste of biodiesel manufacturing.

## Background

Biodiesel is a mixture of fatty acids (FAs) methyl esters obtained from the trans-esterification of triacylglycerols (TAGs). It is produced mostly from plant oils and is regarded as a major resource to face high energy prices and potential depletion of fossil oils reservoirs [1]. Microbial lipids represent a valuable alternative feedstock for biodiesel production and heterotrophic oleaginous microorganisms gave rise to significant attention as lipid producers. Oleaginous yeasts, that accumulate relevant amounts of TAGs within intracellular lipid bodies, have been identified and deeply investigated in the genera *Yarrowia*, *Candida*, *Rhodotorula*, *Rhodosporodium*, *Cryptococcus*, and *Lypomyces*, belonging to both Ascomycota and Basidiomycota phyla [2-5]. TAGs form oleaginous yeasts are similar in composition to plant oils and were successfully exploited in the trans-esterification reaction [6-9]. Moreover, microbial production of TAGs present many advantages (such as low soil consumption, short life cycle, easy scale-up, and low affection by venue, season and climate) that promise to overcome many limitations of plant oils in biodiesel manufacturing [5-7] and provide a novel approach for a more sustainable biofuel production. In biodiesel manufacturing, yeast oleaginous biomass could be dried and subjected to direct methanolysis by acid or alkaline catalysis, but direct transesterification of wet microbial biomass, containing up to 70% water, has been recently described as well [7,10,11].

The utilization of microbial lipids for biodiesel production is economically feasible only if oleaginous microbes are cultured with inexpensive substrates (e.g. the by-products or wastes of other processes) in processes yielding high lipid and biomass concentration and exhibiting adequate yield and productivity. In this perspective, crude glycerol is a very promising raw material, because it is the costless residual of biodiesel production, consisting of a mixture of glycerol (65–85%, w/w), methanol, soaps, and minerals [12], that may be valorized in lipid production. Thus, cultivation of oleaginous yeasts in glycerol-based media is attracting great interest and natural biodiversity is increasingly explored to identify novel oleaginous species using this carbon source for growth and lipid production [13-17]. The process conditions leading to improved lipid production rate and cellular lipid content have been extensively investigated, too. Nutrient imbalance in the culture medium is known to trigger lipogenesis in oleaginous yeasts, the most efficient condition being the occurrence of nitrogen limitation in presence of an excess of carbon source [2,18,19]. Lipid production from glycerol has been studied mostly with batch fermentations, but fed-batch processes are increasingly reported as achieving the highest cell density, lipid production, and substrate conversion [20-22].

In this study, 33 environmental yeasts were screened for the ability to grow and produce intracellular lipids in a glycerol based medium. Due to purification costs, refined glycerol is not feasible for culturing oleaginous microbes and crude glycerol has to be used. However, pure glycerol was used to enable reproducibility of data and medium composition, because the composition of crude glycerol is greatly variable depending on the feedstock, the type of catalysis and the conditions of trans-esterification reaction [23,24]. The best performing strain was selected for deeper investigation of lipid production and for the development of laboratory-scale fed batch process.

## Results and discussion

### Screening for oleaginous yeasts and selection of *C. freyschussii* ATCC 18737

The utilization of glycerol to produce microbial TAGs is a promising strategy enabling a valuable re-utilization of the major waste product of biodiesel manufacturing. Over 40 oleaginous yeast species are known, but the list is lengthening since TAGs-producers have been increasingly searched among environmental yeast isolates, the metabolic potential of which being still understudied. For most of them, however, the ability to accumulate lipids has been assessed in glucose-based media [2,25], without an exhaustive survey on the oleaginicity on glycerol.

In the present study, 33 ascomycetous and basidiomycetous yeast strains belonging to the species *Candida castellii*, *Candida freyschussii*, *Candida maltosa*, *Candida sake*, *Cryptococcus gilvescens*, *Kluyveromyces bacillosporus*, *Kluyveromyces lactis*, *Kluyveromyces lodderae*, *Kluyveromyces marxianus*, *Pichia farinosa*, *Rhodotorula laryngis*, *Saccharomyces boulardii*, *Saccharomyces cariocanus*, *Saccharomyces castellii*, *Saccharomyces cerevisiae*, *Saccharomyces dairenensis*, *Saccharomyces exiguous*, *Saccharomyces spencerorum*, and *Zygosaccharomyces rouxii* were screened for the ability to grow and accumulate intracellular lipids at 30°C in GMY medium containing 40 g/L glycerol (Table 1). A glucose-based GMY medium containing 40 g/L glucose was previously used to screen a set of cold-adapted environmental yeasts [26], with the aim to investigate their ability to produce lipids. The same concentration of the carbon source was maintained in the present study expecting that the carbon/nitrogen imbalance (40 g/L glycerol with 3 g/L yeast extract) was high enough to enable production of storage lipids if the yeasts were oleaginous.

**Table 1:** Screening of yeast strains for growth and lipid production in GMY medium containing 40 g/L glycerol

**Strain**	**Consumed glycerol g/L (%)**	**DW g/L**	**Lipids g/L**	**Y**_**L/S **_**%**	**Y**_**L/X **_**%**
*C. castellii* ATCC 22945	3.2 (8)^a^	1.5	0.1^a^	3^a^	6.5
*C. freyschussii* ATCC 18737	40.0 (100)^b^	11.9	3.2	7.9^b^	26.4^a^
*C. maltosa* ATCC 20275	4.7 (12)^a^	5.77^a^	0.58^b^	12.4^c^	10.1^b^
*C. sake* ATCC 28138	38.8 (97)^b^	6.2^a^	0.27^c^	0.7	4.4^c^
*Cr. gilvescens* DBVPG 4714	*n.g.*				
*Cr. gilvescens* DBVPG 4720	*n.g.*				
*Cr. gilvescens* DBVPG 4722	*n.g.*				
*Cr. gilvescens* DBVPG 4803	*n.g.*				
*Cr. gilvescens* DBVPG 4714	*n.g.*				
*K. bacillosporus* ATCC 200960	36.4 (91)^b^	5.3^a^	0.85^d^	2.3^d^	16
*K. lactis* DBVPG 6969	12.4 (31)^c^	4.42^b^	0.6^b^	4.8^e^	13.6^d^
*K. lodderae* ATCC 6308	15.6 (39)	4.11^b^	0.79^d^	5^e^	19.2^e^
*K. marxianus* ATCC 200963	26.4 (66)	5.97^a^	0.33^c^	1.2	5.5
*K. marxianus* DBVPG 6854	7.6 (19)	6.4^a^	0.26^c^	3.4^a^	4.1^c^
*K. marxianus* CBS 7894	12.0 (30)^c^	5.74^a^	0.25^c^	2.1^d^	4.4^c^
*P. farinosa* CBS 185	40.0 (100)^b^	9.47	1.65^f^	4.1^e^	17.4
*R. laryngis* DBVPG 4772	5.4 (13)^a^	2.72^d^	0.6^b^	11.6^c^	22.1^e^
*R. laryngis* DBVPG 4765	9.2 (23)^d^	3.82^b^	0.75^d^	8.2^b^	19.6^e^
*S. boulardii* H	8.4 (21)^d^	4.7^b^	0.69^b^	8.2^b^	14.7^d^
*S. castellii* ATCC 76901	*n.g.*				
*S. cerevisiae* DBVPG 6036	32.8 (82)	2.06^d^	0.69^b^	2.1^d^	33.5^f^
*S. cerevisiae* DBVPG 6861	13.2 (33)^c^	4.3^b^	1.16^f^	8.9^f^	27^a^
*S. cerevisiae* L17	10.4 (26)^c^	6.18^a^	0.82^d^	7.9^b^	13.3^d^
*S. cerevisiae* ATCC 26785	6.6 (16)^a^	4.87^b^	0.61^b^	9.6^f^	12.5^d^
*S. cerevisiae* ATCC 2345	7.2 (18)^d^	4.18^b^	0.42^e^	5.9^g^	10^b^
*S. cariocanus* ATCC 201563	*n.g.*				
*S. dairenensis* DBVPG 6357	*n.g.*				
*S. exiguus* L10	*n.g.*				
*S. spencerorum* ATCC 200069	39.2 (98)^b^	6.42^c^	2.4	6.1^g^	37.4
*S. spencerorum* ATCC 60635	37.2 (93)^b^	4.86^b^	1.57^f^	4.2^e^	32.3^f^
*Z. rouxii* ATCC 52711	*n.g.*				
*Z. rouxii* L21	*n.g.*				
*Z. mellis* CBS 1091	14 (35)^c^	7.05^c^	0.69^b^	4.9^e^	9.8^b^

The yeasts were cultured for 120 h in GMY medium containing 40 g/L glycerol. The amount of glycerol consumed during incubation, final biomass dry weight (DW) and lipids, and lipid/glycerol and lipid/biomass yield coefficients (Y_L/S_ and Y_L/X_) are reported. Values are means, n = 3, relative SD always < 7%. Within each column, means are significantly different (*P* < 0.05), unless they have a common letter. *n.g.* indicates strains that did not grow.

The medium proved to be satisfactory for the identification of some yeasts capable of growth, efficient substrate consumption, and higher lipid accumulation. Twenty-three strains of 33 exhibited some growth in 120 h, consuming at least 2 g/L glycerol (5% of initial concentration) and yielding a DW in the range from 1.5 to 11.9 g/L. Among them, only *C. freyschussii* ATCC 18737, *K. bacillosporus* ATCC 200960, *P. farinosa* L19, *S. spencerorum* ATCC 200069, and *S. spencerorum* ATCC 60635 consumed more than 90% glycerol and were also able to accumulate lipids, based on the appearance of visible intracellular lipid bodies positive to Sudan Black staining. The highest amount of intracellular lipids were produced by *C. freyschussii* ATCC 18737, *P. farinosa* L 19, and *S. spencerorum* ATCC 200069, which yielded 3.2, 1.6, and 2.4 g/L intracellular lipids, corresponding to 26.4, 17.4, and 37.4% of dry biomass, respectively (Table 1). *C. freyschussii* ATCC 18737, which exhibited the highest biomass yield and lipid production (P < 0.05) in terms of lipid concentration and glycerol conversion to lipids (Y_L/S_) (Table 1), was selected for further investigation.

It is noteworthy that, to handle a reproducible and controllable cultural medium, pure glycerol was used first, without considering any potential effect of the impurities occurring in crude glycerol. Beyond certain levels, crude glycerol impurities (in particular alcohols, soaps, and metals) are known to negatively affect or inhibit microbial growth [27]. However, certain oleaginous fungi were reported to give higher biomass and/or lipid yield with crude glycerol (e.g. from alkali- or lipase-catalyzed biodiesel production) compared with pure glycerol [15,24,28-32]. In fact, equivalent amounts of crude glycerol result in lower osmotic stress compared to pure glycerol and bear nutrients that may enhance growth (i.e. the salts) or may be used for biomass and/or lipid production (e.g. free fatty acids) [13,31]. The main inhibitive effect of crude glycerol is ascribed to methanol, the removal of which through an evaporation process may be necessary for utilization in cultural media [24,27,30,32]. Among the other impurities, inorganic salt, glycerides, and soaps positively affected both biomass and lipid production, while methyl esters did not exert any inhibition [32].

Subsequent studies will follow based on these evidences, in order to challenge *C. freyschussii* ATCC 18737 on crude glycerol and to evaluate the effect of the single impurities coming from specific biodiesel manufactures. A positive outcome of this approach would render *C. freyschussii* ATCC 18737 a good candidate strain for industrial lipid production, like *Cryptococcus curvatus*, *Mortierella isabellina*, *Rhodotorula glutinis, and Yarrowia lipolytica*[19,20,31,33]*.*

### Effects of the C/N ratio on lipid production

**Figure 1:**
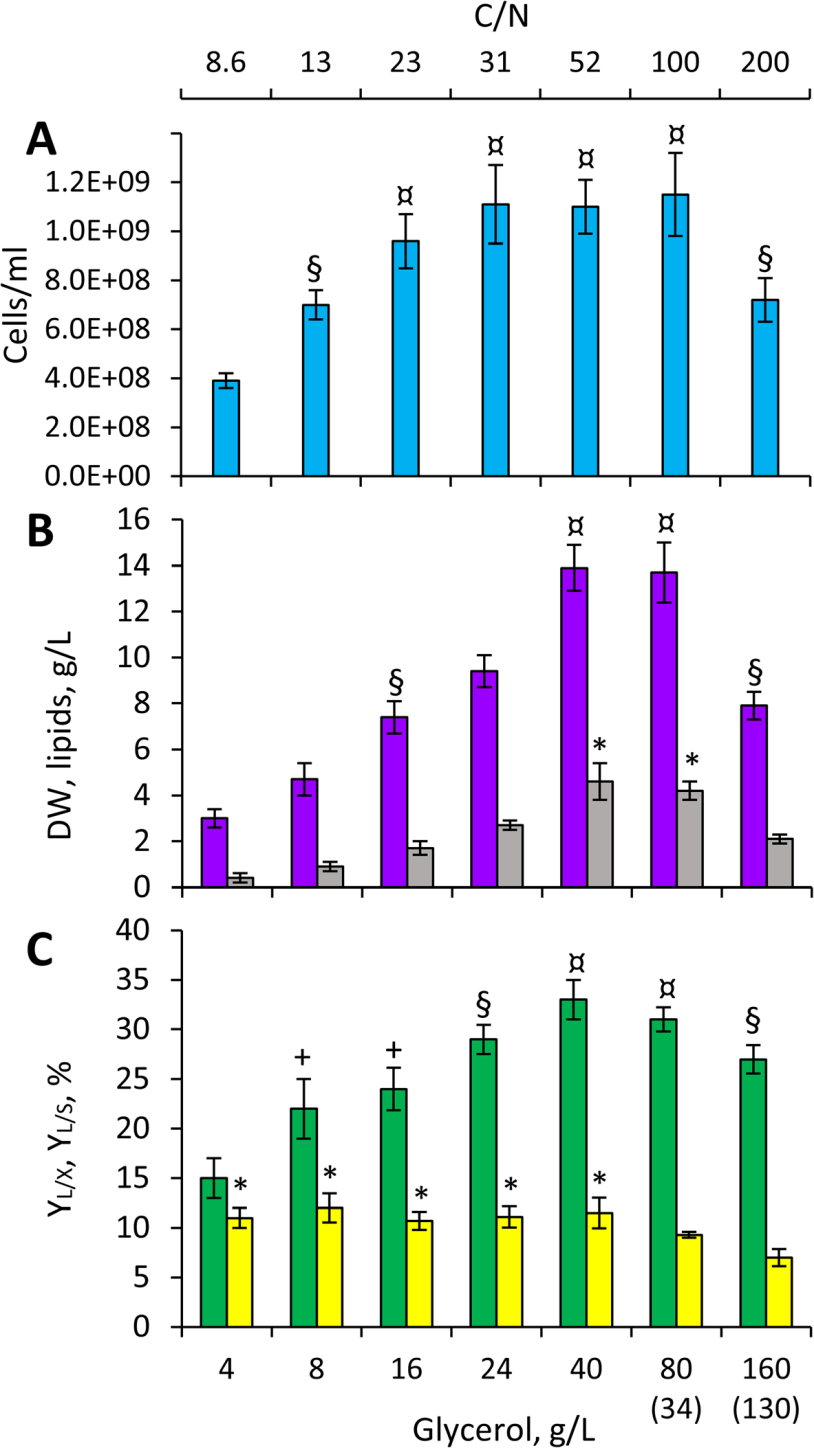
**Effect of the C/N ratio on growth and lipid production of *C. freyschussii *ATCC 18737.***C. freyschussii* ATCC 18737 was cultured in GMY medium containing 3 g/L yeast extract and different glycerol concentration, in order to obtained different C/N ratios. Biomass was harvested and lipid content was analyzed at the latest after 72 h, unless glycerol was depleted earlier. Residual glycerol, if any, is reported in brackets. **A**, final cell count (cyan); **B**, final concentrations: biomass (purple) and lipids (grey); **C**, yield coefficients: lipid/biomass (Y_L/X_, green) and lipid/glycerol (Y_L/S_, yellow). Values are means ± SD, n = 3. Within each series, means are significantly different (P < 0.05), unless they have a common superscript.

**Figure 2:**
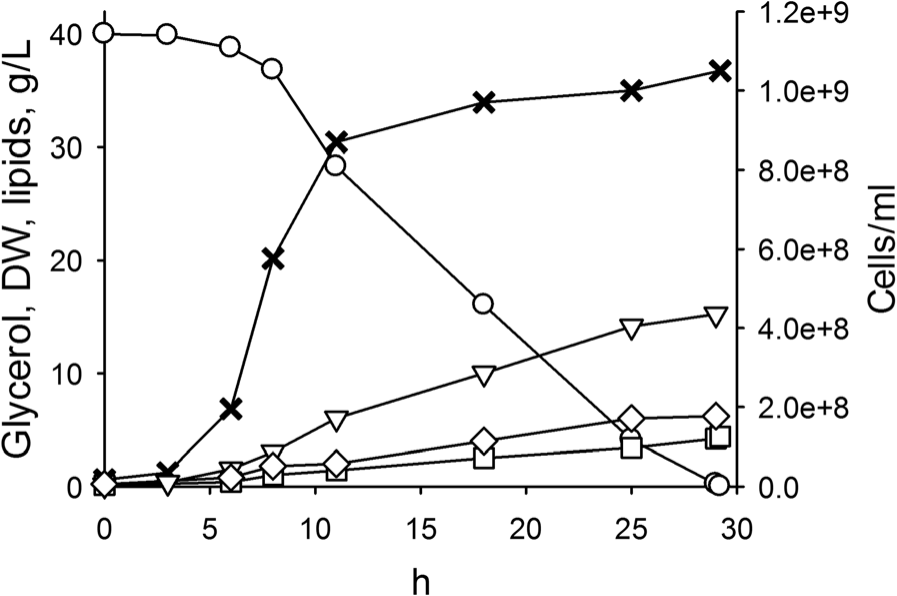
**Time-course of growth and lipid production in batch culture of *C. freyschussii *ATCC 18737.** The strain was cultured in GMY medium, containing 40 g/L glycerol. The multiplication of cells finished after 10 h due to nitrogen limitation, then the carbon excess was channeled toward accumulation of lipids and carbohydrates. Symbols: glycerol, ○; biomass, ▽; biomass-associated carbohydrates, ◊; biomass-associated lipids, □; cell counts, ×. The experiment was carried out in triplicate. A representative time-course is reported herein.

It is known that a nutrient imbalance in the culture medium, and high C/N ratio in particular, positively affect lipid production by oleaginous species [2,34,35]. Thus, batch cultures of *C. freyschussii* ATCC 18737 were carried out in GMY medium, with 3 g/L yeast extract and glycerol concentrations ranging from 4 to 160 g/L (Figure 1, Table 2). The carbon source run out within the first 30 h if initial glycerol was 40 g/L or less. At least 16 g/L glycerol were necessary to establish nitrogen-limited conditions, as indicated by the final cell counts (Figure 1A). Even though the cells did not further increase due to nitrogen limitation, the increase of initial glycerol up to 40 g/L determined the increase of both final biomass and intracellular lipids (Figure 2A) and positively correlated with lipid/biomass yield (Y_L/X_), indicating that higher amount of the carbon flow was directed toward the synthesis of storage lipid (Figure 1C). Higher glycerol concentrations inhibited the culture and did not determine any further rise in lipid production. In fact, in presence of 80 and 160 g/L glycerol the yeast yielded less biomass and failed to deplete the carbon source in 72 h (Table 2). Therefore, *C. freyschussii* ATCC 18737 exhibited the highest lipid production (4.6 g/L), lipid content of biomass (33%), and lipid volumetric productivity (0.15 g/L/h) with 40 g/L glycerol, during the course of a 30-h batch process. Growth inhibition in presence of high glycerol concentration might be due to osmotic stress and was described also for other yeasts cultured in pure or crude glycerol, with the exception of *Y. lipolytica*[15,36,37]. Thus, the behavior of *C. freyschussii* ATCC 18737 is in line with most fungi, which are inhibited by glycerol at concentration ranging from 40 to 100 g/L [15,20,21].

**Table 2:** **Effect of the C/N ratio on the fatty acids profile of *C. freyschussii *** **ATCC 18737**

**Glycerol**	**C16**	**C16:1**	**C18**	**C18:1**	**C18:2**	**C18:3**	**C20**	**Total C16**	**Total C18**	**UI**
g/L	%	%	%	%	%	%	%	%	%	
4	17.5^a^	11.9	6.1	32.5	24.7	6.9	0.4^a^	29.4	70.2	1.15^a^
8	17.3^a^	18.9^a^	4.0	39.3^a^	16.7	3.6	0.2^a^	36.2^a^	63.6^a^	1.02^a,b^
16	17.1^a^	24.6^b^	2.7^a^	41.7^a^	11.9^a^	2.0	0	41.7^b^	58.3^b^	0.96^b^
24	16.8^a^	24.2^b^	2.3^a^	44	11.1^a^	1.6	0	41.0^b^	59.0^b^	0.95^b^
40	16.6^a^	19.9^c^	3.5^b^	48.8^b^	10.5^a^	0.7^a^	0	36.5^a^	63.5^a^	0.92^b^
80	16.9^a^	20^c^	2.5^a^	47.4^b^	12.2^a^	1.0^a^	0	36.9^a^	63.1^a^	0.95^b^
160	17.1^a^	19.8^c^	3.5^b^	46.9^b^	11.2^a^	1.3^a^	0	37.7	62.3	0.92^b^

*C. freyschussii* ATCC 18737 was cultured in GMY medium containing 3 g/L yeast extract and different glycerol concentration, in order to obtained different C/N ratios. Biomass was harvested for lipid analysis at the latest after 72 h, unless glycerol was depleted earlier. Final fatty acids profile are reported. Values are means, n = 3, SD always < 3%. Within each column, means are significantly different (*P* < 0.05), unless they have a common letter.

### Lipid composition and predicted biodiesel properties

The ^1^H-NMR spectrum of the lipids extracted from nitrogen limited cultures of *C. freyschussii* ATCC 18737 indicated that the extracts were mostly composed of TAGs and did not contain detectable free fatty acids or other polar lipids. In fact, all the signals in ^1^H-NMR spectra fitted the literature spectra of plant oils. The triplet signal of α-CH_2_ at 2.31 ppm suggested the presence only of esters and the absence of free FAs [38]. Regardless of the C/N ratio, the FAs profile of *C. freyschussii* ATCC 18737 (Table 2) was dominated by the C18 ones followed by the C16 (58.3 to 70.2% the former, 29.4 to 41.7% the latter). Myristic acid and the FAs longer than C18 were always negligible. With the exception of palmitic acid, always accounting for approx. 17% (*P* > 0.05), glycerol concentration affected the relative abundance of each FA in nitrogen limited media, compared with carbon limited ones (Table 2). The differences due to glycerol concentration were minor or negligible among nitrogen-limited cultures. Comparison of fatty acids composition in cultures grown at 4 or 40 g/L revealed that in the passage from carbon to nitrogen limited media saturated FAs decreased from 23.6 to 19.4%, due to the slight reduction of stearic acid from 6.1 to 2.7% (*P* < 0.05). Monounsaturated FAs (C16:1 + C18:1) were always the most abundant and increased from 44.4 to 68.7% (*P* < 0.05), principally due to oleic acid, always the most abundant FA, which passed from 32.5 to 48.8% (*P* < 0.05). The polyunsaturated FAs decreased from 33.6 to11.2%, linoleic and linolenic acid dropping from 24.7 and 6.9% to 10.5 and 0.7% with 4 and 40 g/L glycerol, respectively.

Differences in FA profile are known to occur, depending on medium composition [4,39], but have not been conclusively explained yet. Lipid droplets have been recently recognized as highly dynamic organelles, acting as storage depots of neutral lipids and playing a central role in the homeostasis of fatty acids in both triacylglycerols and membrane phospholipids. The observation that different C/N ratios cause changes in fatty acids composition likely arise from different metabolic states which affect the FA biosynthesis in order to meet different cellular needs (e.g. the phospholipids for cellular membranes, required during growth, or neutral storage lipids in nitrogen limited cultures).

The lipids produced by *C. freyschussii* ATCC 18737 have similar fatty acid composition to that of plant oil indicating their potential use as biodiesel feedstock [40]. In order to establish if they are suitable for biodiesel production, the physicochemical properties which depend on the FA profile of the feedstock (density, kinematic viscosity, flash point temperature, heating value, and saponification, iodine, and cetane numbers) were preliminary calculated using database values of individual pure fatty acids methylesters and predictive equations (Table 3) [41]. Rapeseed and palm oil, two major plant feedstocks, were analyzed with the same equation and compared [40]. The putative physicochemical properties of a biodiesel composed of FAME from *C. freyschussii* ATCC 18737 lipids are in line with those of rapeseed and palm biodiesels, and are expected to meet the European and American standards (EN 14214 and ASTM D6751, respectively).

**Table 3:** **Prediction of methylesters properties, based on fatty acids profile *C. freyschussii *** **ATCC 18737**

	***C. freyschussii *** **ATCC 18737**	**Rapeseed oil**	**Palm oil**	**US standard ASTM D6751**	**EU standard EN 14214**
Density at 15°C, g/L	878	876	852	-	860 - 900
Kinematic viscosity at 40°C, mm^2^/s	4.27	4.18	4.32	1.9 - 6.0	3.5 - 5.0
Saponification number	196	189	192	-	-
Iodine number	81.0	115.1	52.2	-	≤ 120
Cetane number	54.0	46.7	59.2	47 - 65	≥ 51
Higher heating value, MJ/Kg	40.2	40.0	40.8	-	-
Flash point, °C	117.5	106.6	144.5	≥ 93	≥ 101
C18:3, %	0.7	8.2	0.2	-	≤ 12
PUFAs with ≥ 4 double bonds, %	0			-	≤ 1

The main physicochemical properties of methylesters were predicted, based on the FA profile of *C. freyschussii* ATCC 18737 and typical composition of rapeseed and palm oils. Values from American and European standards are also reported.

### Batch cultures

*C. freyschussii* ATCC 18737 was cultured in bioreactor in order to evaluate the kinetics of growth and lipid production in nitrogen limited GMY medium containing 40 g/L glycerol. Its behavior was similar to that observed with other oleaginous fungi cultured in nitrogen limited media [2,34,42], since growth and lipogenesis proceeded through two consecutive phases (Figure 2). Exponential balanced growth occurred during the first 11 h, yielding the increase of both cell counts and biomass dry weight (up to 1.0e + 09 cells/ml and 6.1 g/L, respectively). Accumulation of storage lipids took place at some extent even during the growth phase, since 1.1 g/L lipids were produced and reached the 18% of biomass weight, with a mean rate of 0.10 g/L/h. At the end of the growth phase, total cellular carbohydrates accounted for 1.8 g/L, i.e. the 30% of biomass.

The growth phase took place at the expenses of only approx. 12 g/L glycerol, then the remaining glycerol continued to be consumed and get exhausted after 30 h of cultivation. In this latter phase, the increase of cell counts was minor, due to nitrogen limitation. On the contrary, the consumption of the excess of glycerol was accompanied by the increase of biomass weight (up to 15.2 g/L), due to the synthesis and accumulation of both carbohydrates and lipids. Unlike other oleaginous yeasts, citrate or other low molecular weight metabolites were never produced at detectable levels [42]. Cellular carbohydrates increased up to 6.1 g/L, accounting for the 40% of dry biomass. Lipid production occurred with a rate of 0.18 g/L/h and yielded 4.7 g/L lipids, which accounted for the 32% of biomass after 30 h. Thus, the whole batch process performed a volumetric productivity of lipids of 0.16 g/L/h with a lipid/glycerol yield (Y_L/S_) of 12%. The maximum theoretical lipid yield from glycerol is approx. 30% w/w [18], but reported Y_L/S_ values generally range between 8 and 12%, one exception being the fungus *M. isabellina* that performed lipid production from crude glycerol with a yield of 15% [33]. In this context, the performance of *C. freyschussii* ATCC 18737 is higher than that of most oleaginous yeasts reported in literature. Yet, glycerol conversion into lipids remains quite low and a relevant amount of the carbon flux is drained into storage carbohydrates.

### Fed-batch processes

**Figure 3:**
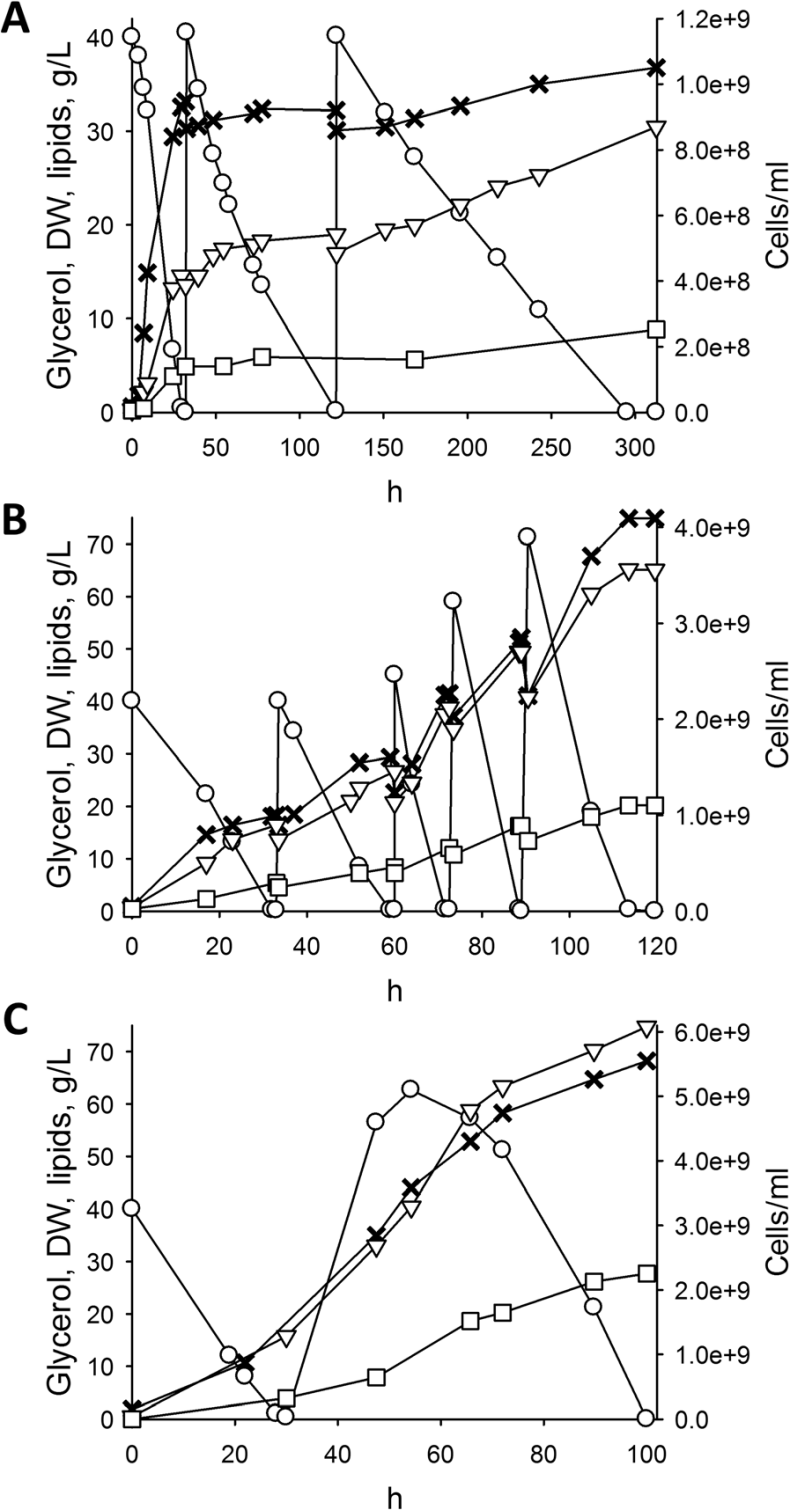
**Time-course of growth and lipid production by in fed-batch cultures of *C. freyschussii *ATCC 18737.** Fed-batch cultures were utilized in order to extend the lipogenic phase without incurring in substrate inhibition. Three approaches were tested: A, pulsed feeding of concentrated glycerol; B, pulsed feeding of C-GMY medium; C, continuous feeding of C-GMY medium. Symbols: glycerol, ○; biomass, ▽; biomass-associated lipids, □; cell counts, ×. The experiments were carried out in triplicate. Representative time-courses are reported herein.

**Figure 4:**
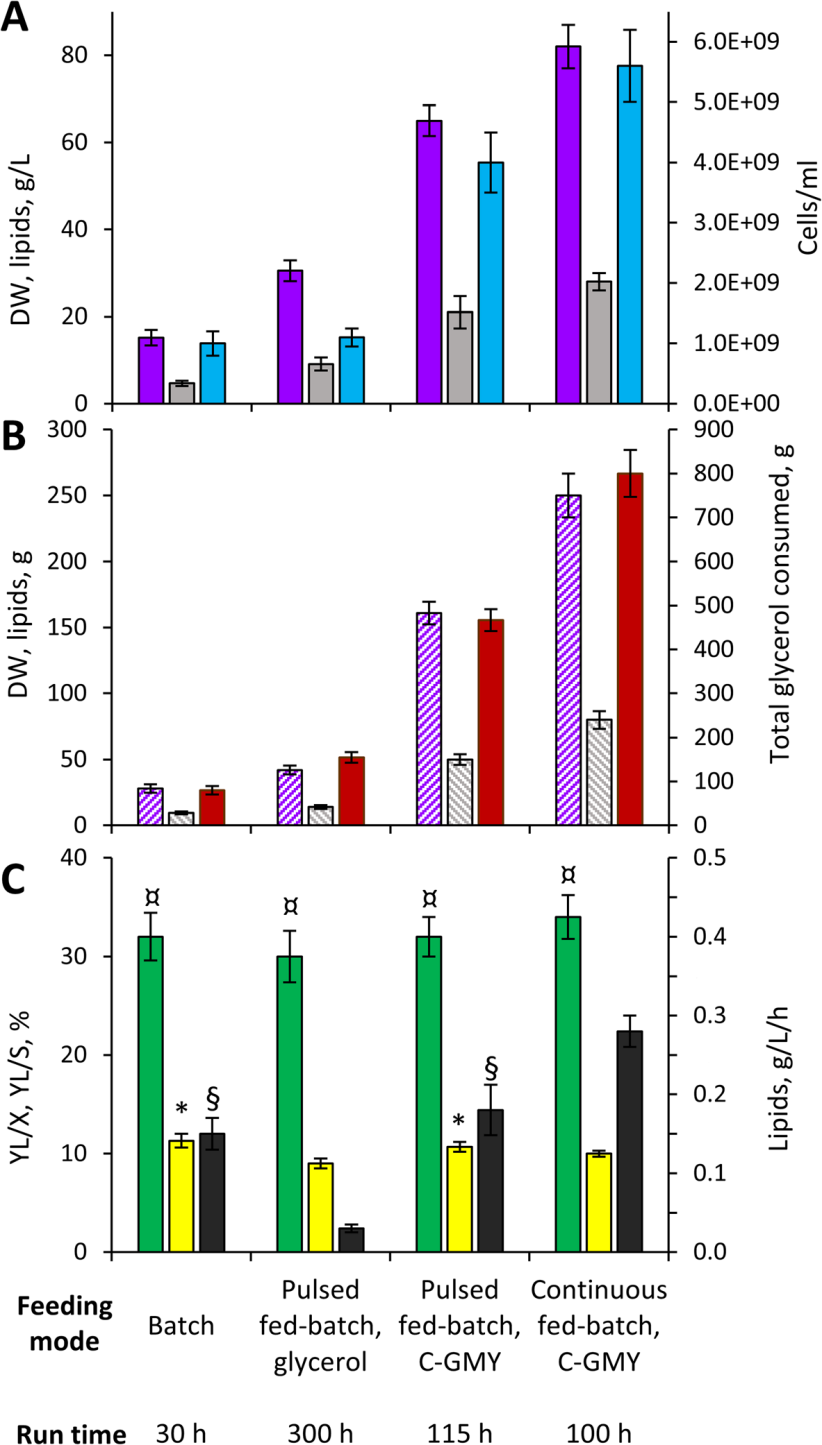
**Fermentation performance under various operation modes.** Mean performances of batch and fed-batch processes are summarized. **A**, final concentration of dry biomass (purple), lipids (grey), and cells (cyan). **B**, total mass of biomass generated (dashed purple), lipids generated (dashed grey), and glycerol consumed (red), calculated considering the volume changes and the contribution of both the feeding and the sample withdrawal. **C**, lipid/biomass (Y_L/X_, green), lipid/glycerol (Y_L/S_, yellow), and lipid productivity (grey). Within each series, values are significantly different (*P* < 0.05, n = 3), unless they have a common superscript.

Since the extent of the carbon excess positively affected lipid production, but high glycerol concentration inhibited growth, fed-batch cultures have be utilized to furnish additional glycerol without incurring in strain inhibition [20,21,31]. Thus, three different feeding strategies were attempted with the aim to prevent growth inhibition and extend the lipogenic phase, improving lipid production (Figure 3, Figure 4).

In the first fed-batch experiment, after 30 h batch-wise cultivation in GMY medium, *C. freyschussii* ATCC 18737 was given two consecutive pulses of concentrated glycerol to restore 40 g/L glycerol as soon as the carbon source was depleted (Figure 3A). All the glycerol fed was consumed in 300 h, yielding a slight increase of cell counts accompanied by a greater increase of biomass concentration. At the end of the process 30.5 g/L biomass were achieved, 9.1 g/L of which were cellular lipids (Y_L/X_ = 30%). Despite the greater amount of lipids produced, the volumetric productivity was lower (0.03 g/L/h) compared to the batch process, because glycerol consumption rate progressively decreased, causing the production rate to decline as well (Figure 4).As glycerol pulses did not improve the productivity, different feeding approaches were tried out, consisting in the addition of a concentrated medium, where both carbon and nitrogen sources were both 20-fold more concentrated, compared with GMY medium (hereinafter named C-GMY). When 4 repeated pulses of C-GMY medium were given (Figure 3B), the cell counts continued to increase because the culture was provided with the nitrogen source, thus a high-cell density culture of 4.0e + 09 cells/ml and 65 g/L biomass was obtained in 115 h. Glycerol was consumed with improving rates as the culture became more dense, thus enabling the feeding of progressively more abundant pulses without incurring in any inhibition. The culture generated 20 g/L lipids, accounting for the 32% of biomass, with a mean volumetric productivity of 0.18 g/L/h (Figure 4).

When C-GMY was continuously fed (Figure 3C), the culture was provided with 5.5 g/L/h of glycerol, approx. 3-fold higher than the highest consumption rate registered during the batch phase (1.8 g/L/h). The continuous feeding started at 30 h of cultivation. It resulted in initial glycerol accumulation at the rate of 3.4 g/L/h, with a concurrent increase of cell density and biomass concentration, which caused glycerol consumption rate to increase as well. As a result of the progressive increase in consumption rate, glycerol reached the highest concentration when the consumption rate became equal to the feeding one (62 g/L, after 24 h of feeding), then it decreased as the consumption rate further increased above 5.5 g/L/h. After 70 h of feeding, the culture contained < 0.3 g/L glycerol, 5.6e + 09 cells/ml, and 82 g/L biomass, 28 g/L of which were cellular lipids (Y_L/X_ = 34%). In these conditions, lipids were produced with the mean volumetric productivity of 0.28 g/L/h (Figure 4).

The data herein presented confirm that fed-batch fermentations are adequate to prevent inhibition at high glycerol concentrations, thus enabling a relevant improvement of process performance [20,21,31]. In particular, cultivation of *C. freyschussii* ATCC 18737 in fed-batch mode succeeded in increasing both lipid concentration and volumetric productivity to remarkable high levels, in particular with the continuous feeding of C-GMY. This achievement was mostly due to the increase of cell density and biomass concentration, both contributing at the improvement of lipid production rate and lipid concentration in the culture. On the other hand, feeding a concentrated medium with the same C/N ratio did not influence carbon allocation, compared with the batch process, thus the lipid content of biomass and lipid/glycerol yield remained both unaffected. Likewise, storage carbohydrate accumulation also remained unaffected, since the carbohydrate content within biomass always ranged between 40 and 43% without any significant difference among the processes.

Compared with previous studies describing batch and fed-batch processes using oleaginous yeasts for lipid production from pure and/or crude glycerol, the best fed-batch process of *C. freyschussii* ATCC 18737 yielded greater lipid concentration and volumetric productivity. Higher values (29 g/L lipids, 0.6 g/L/h) were obtained only in fed-batch process of *Cr. curvatus* with pure glycerol [20]. Conversely, diverse oleaginous fungi exhibited higher Y_L/X_ than *C. freyschussii* ATCC 18737 in batch and fed-batch processes in glycerol-based media [21,31]. As an example, the biomass of *R. glutinis* contained lipids up to the 61% during fed-batch cultivation with crude glycerol, but overall lipid concentration and volumetric productivity were lower (6.1 g/L lipids, 0.09 g/L/h) [31]. Overall, lipid production *C. freyschussii* ATCC 18737 has potential to be profitable at the same extent (or more) compared with other processes described with oleaginous fungi. In particular, the fed-batch processes herein described present higher lipid production and productivity, compared with most processes proposed in literature, albeit the process needs to be adapted to the utilization of crude glycerol, much cheaper than pure glycerol.

## Conclusions

This study explored lipid production from glycerol in 33 yeast strains and focused on *C. freyschussii* ATCC 18737, which exhibited the highest yield. Lipid production was positively affected by the increase of C/N ratio, but growth was inhibited by high glycerol concentration. Fed-batch cultivation succeeded in preventing substrate inhibition and in achieving a high cell-density culture, resulting in remarkable high lipid production and volumetric productivity (28 g/L and 0.28 g/L/h, respectively). The lipids produced by *C. freyschussii* ATCC 18737 have similar fatty acid composition to that of plant oil indicating their potential use as biodiesel feedstock. Predicted physicochemical properties of a biodiesel produced from the lipids *C. freyschussii* ATCC 18737 are in line with those of rapeseed and palm biodiesels, and are expected to meet the European and American standards. Therefore, *C. freyschussii* ATCC 18737 could be considered an interesting microorganism for utilization in biofuel industry. Cultivation of this yeast in media containing crude glycerol should be further investigated in order to evaluate whether it might find application in the valorization of the waste of biodiesel manufacturing.

## Methods

### Strains and culture conditions

Thirty-three environmental yeast strains used in this study were obtained from ATCC (Manasses, VA, USA), CBS (Utrecht, the Netherlands), and DBVPG (Industrial Yeasts Collection, University of Perugia, Italy), or from our own collection. To evaluate lipid production, aerobic cultures were carried out at 30°C in flasks of the carbon rich GMY medium that contained 40 g/L glycerol, 8 g/L KH_2_PO_4_, 0.5 g/L MgSO_4_ · 7H_2_O, 3 g/L yeast extract (BD Difco), and 0.1 ml PTM1 microelements solution [26,43]. To investigate the effect of the C/N ratio, batch experiments were carried out in GMY medium containing 4, 8, 16, 24, 40, 80, and 160 g/L glycerol. Biomass was harvested and lipid content was analyzed at the latest after 72 h, unless glycerol was depleted earlier. All chemicals were obtained from Sigma-Aldrich (Steinheim, Germany).

The lipid/biomass yield (Y_L/X_) was calculated as the grams of lipid produced per gram of biomass and corresponded to the lipid content of biomass. The lipid/glycerol yield (Y_L/S_) was calculated as the grams of lipid produced per grams of glycerol consumed.

### Batch and fed-batch processes

Batch experiments were carried out in a benchtop bioreactor (Labfors, Infors, Bottmingen, Switzerland) with 2 L of GMY medium, inoculated 5% v/v with a 24-h seed culture grown in GMY containing 4 g/L glycerol. The culture was kept at 30°C and aerated with 1 v/v/min air; stirring was regulated in the range from 150 to 900 rpm to keep the DOT at 20%.

Fed-batch experiments were initiated batchwise in 2 L of GMY medium containing 40 g/L glycerol. As the culture entered into the stationary phase, one of the following feeding modes was applied: i) pulses of 400 g/L glycerol, repeatedly given to reinstate 40 g/L glycerol whenever the carbon source was exhausted; ii) pulses of C-GMY medium (containing 800 g/L glycerol,60 g/L yeast extract, 8 g/L KH_2_PO_4_, 0.5 g/L MgSO_4_ · 7 H_2_O, and 0.1 ml/L PTM1 solution), repeatedly given to reinstate 40 g/L glycerol; iii) continuous feeding C-GMY medium, to provide 5.5 g/L/h glycerol. Samples were periodically collected to monitor glycerol, growth, and the amount of cellular lipids and carbohydrates.

### Chemical analysis

Glycerol concentration was analyzed using HPLC device (Agilent technologies, Waldbronn, Germany) equipped with refractive index detector (RID) and Aminex HPX-87 H ion exclusion column. Isocratic elution was carried out at with 5 mM H_2_SO_4_ at 0.6 ml min^-1^. Biomass concentration (dry weight, DW) was determined gravimetrically using pre-weighed 0.2 μm filters; cell counts were quantified in a Bürker chamber at the optical microscope. Cellular carbohydrates were quantified by reacting yeast biomass with anthrone reagent [44]. To evaluate the presence of cellular lipid bodies, yeast cells were pelleted, incubated for 15 min in a filtered solution of Sudan Black B (5 g/L in ethylene glycol), and observed at the optical microscope.

Cellular lipids were extracted from lyophilized biomass with chloroform: methanol mixture (2:1, v/v) and were determined gravimetrically [26]. The lipid extract was subjected to ^1^H-NMR spectroscopy to evaluate triacylglycerols, polar lipids, and free fatty acids [38]. To determine the fatty acids (FA) composition, the corresponding methyl-esters were generated and analyzed by GC-MS. The lipid extract was diluted 1:20 (w/v) in ethyl ether, then 100 μL of this solution were mixed with 900 μL of ethyl ether containing 1 g/L glyceryl triundecanoateas, the internal standard, and 50 μL of sodium methanoate (3.3 M in methanol). 30 μL of acetic acid were added after 10 min at room temperature, then the mixture was clarified by centrifugation (4000 g, 4 min) and 1 μL was injected into a quadrupole GC-MS system (HP5890 HP5972, Agilent Technologies) equipped with CP–Select CB column for FAME (Varian). The injection temperature was 270°C; the oven temperature was programmed with a 2.5°C/min increase from 160°C to 250°C and 20 min isotherm at 280°C. Peak areas in the total ion chromatograms were used to determine their relative amounts. To aid the analysis of lipid composition, the unsaturation index (UI) was calculated as the number of the double bonds of each fatty acids multiplied by its relative amount.

### Prediction of biodiesel properties

The physicochemical properties of biodiesel (i.e. the density, kinematic viscosity, flash point temperature, heating value, and saponification, iodine, and cetane numbers), that depend on the FA profile, were calculated using predictive equations (Additional file 1: Table S1), based on reported properties of individual pure fatty acid methyl esters [41,45-48].

### Statistical analysis

All values are means of three separate experiments. Differences in means were analyzed using ANOVA for independent measures, followed by Tukey post hoc comparisons (SPSS version 20, IBM, Armonk, USA). Differences were considered statistically significant for *P* < 0.05.

## Competing interests

The authors declare that they have no competing interests.

## Authors’ contributions

SR performed most of the experimental work. AL performed lipid extraction and GC-MS analysis. MMB and TR contributed to the design of fed-batch fermentation experiments and data interpretation. AA and MR conceived the study and drafted the manuscript. All authors read and approved the final manuscript.

## Supplementary Material

Additional file 1: Table S1 Calculation of biodiesel physicochemical properties from the composition of the fatty acids methylesters. Click here for file

## References

[B1] Zinoviev S Müller-Langer F Das P Bertero N Fornasiero P Kaltschmitt M Centi G Miertus S Next-generation biofuels: survey of emerging technologies and sustainability issues ChemSusChem 2010 3 1106 1133 10.1002/cssc.201000052 20922754

[B2] Ageitos J Vallejo J Veiga-Crespo P Villa T Oily yeasts as oleaginous cell factories Appl Microbiol Biotechnol 2011 90 1219 1227 10.1007/s00253-011-3200-z 21465305

[B3] Li Q Du W Liu D Perspectives of microbial oils for biodiesel production Appl Microbiol Biotechnol 2008 80 749 756 10.1007/s00253-008-1625-9 18690426

[B4] Amaretti A Raimondi S Sala M Roncaglia L De Lucia M Leonardi A Rossi M Single cell oils of the cold-adapted oleaginous yeast *Rhodotorula glacialis* DBVPG 4785 Microb Cell Fact 2010 9 73 20863365 10.1186/1475-2859-9-73 PMC2955590

[B5] Rossi M Amaretti A Raimondi S Leonardi A Stoytcheva M, Montero G Getting Lipids for Biodiesel Production from Oleaginous Fungi Biodiesel - Feedstocks and Processing Technologies 2011 Rijeka, Crotia: InTech - Open Access Publisher 71 92

[B6] Azócar L Ciudad G Heipieper H Navia R Biotechnological processes for biodiesel production using alternative oils Appl Microbiol Biotechnol 2010 88 621 636 10.1007/s00253-010-2804-z 20697706

[B7] Liu B Zhao ZK Biodiesel production by direct methanolysis of oleaginous microbial biomass J Chem Technol Biotechnol 2007 82 775 780 10.1002/jctb.1744

[B8] Zhu LY Zong MH Wu H Efficient lipid production with *Trichosporon fermentans* and its use for biodiesel preparation Bioresour Technol 2008 99 7881 7885 10.1016/j.biortech.2008.02.033 18394882

[B9] Cheirsilp B Louhasakul Y Industrial wastes as a promising renewable source for production of microbial lipid and direct transesterification of the lipid into biodiesel Bioresour Technol 2013 142 329 337 23747444 10.1016/j.biortech.2013.05.012

[B10] Thliveros P Uçkun Kiran E Webb C Microbial biodiesel production by direct methanolysis of oleaginous biomass Bioresour Technol 2014 157 181 187 24556371 10.1016/j.biortech.2014.01.111

[B11] Cui Y Liang Y Direct transesterification of wet *Cryptococcus curvatus* cells to biodiesel through use of microwave irradiation Appl Energy 2014 119 438 444

[B12] da Silva GP Mack M Contiero J Glycerol: a promising and abundant carbon source for industrial microbiology Biotechnol Adv 2009 27 30 39 10.1016/j.biotechadv.2008.07.006 18775486

[B13] Easterling ER French WT Hernandez R Licha M The effect of glycerol as a sole and secondary substrate on the growth and fatty acid composition of *Rhodotorula glutinis* Bioresour Technol 2009 100 356 361 10.1016/j.biortech.2008.05.030 18614357

[B14] Duarte SH de Andrade CC Ghiselli G Maugeri F Exploration of Brazilian biodiversity and selection of a new oleaginous yeast strain cultivated in raw glycerol Bioresour Technol 2013 138 377 381 23623430 10.1016/j.biortech.2013.04.004

[B15] Marchand K Lubitz WD Nicol RW Utilization of biodiesel derived crude glycerol by fungal isolates for biomass and single cell oil production J Biobased Mater Bio 2013 7 415 419 10.1166/jbmb.2013.1367

[B16] Juszczyk P Tomaszewska L Kita A Rymowicz W Biomass production by novel strains of *Yarrowia lipolytica* using raw glycerol, derived from biodiesel production Bioresour Technol 2013 137 124 131 23587815 10.1016/j.biortech.2013.03.010

[B17] Kitcha S Cheirsilp B Enhancing lipid production from crude glycerol by newly isolated oleaginous yeasts: strain selection, process optimization, and fed-batch strategy Bioenergy Res 2013 6 300 310 10.1007/s12155-012-9257-4

[B18] Ratledge C Wynn JP Laskin AI, Bennett JW, Gadd GM The Biochemistry and Molecular Biology of Lipid Accumulation in Oleaginous Microorganisms Advances in Applied Microbiology 2002 San Diego: Elsevier Academic Press Inc 51 1–51 10.1016/s0065-2164(02)51000-5 12236054

[B19] Granger LM Perlot P Goma G Pareilleux A Efficiency of fatty acid synthesis by oleaginous yeasts: prediction of yield and fatty acid cell content from consumed C/N ratio by a simple method Biotechnol Bioeng 1993 42 1151 1156 10.1002/bit.260421004 18609663

[B20] Meesters PAEP Wal H Weusthuis R Eggink G Cultivation of the oleaginous yeast *Cryptococcus curvatus* in a new reactor with improved mixing and mass transfer characteristics Biotechnol Tech 1996 10 277 282

[B21] Liang Y Cui Y Trushenski J Blackburn JW Converting crude glycerol derived from yellow grease to lipids through yeast fermentation Bioresour Technol 2010 101 7581 7586 10.1016/j.biortech.2010.04.061 20478702

[B22] Uçkun Kiran E Trzcinski A Webb C Microbial oil produced from biodiesel by-products could enhance overall production Bioresour Technol 2013 129 650 654 23298770 10.1016/j.biortech.2012.11.152

[B23] Chatzifragkou A Papanikolaou S Effect of impurities in biodiesel-derived waste glycerol on the performance and feasibility of biotechnological processes Appl Microbiol Biotechnol 2012 95 13 27 10.1007/s00253-012-4111-3 22581036

[B24] Nicol RW Marchand K Lubitz WD Bioconversion of crude glycerol by fungi Appl Microbiol Biotechnol 2012 93 1865 1875 10.1007/s00253-012-3921-7 22322872

[B25] Tanimura A Takashima M Sugita T Endoh R Kikukawa M Yamaguchi S Sakuradani E Ogawa J Shima J Selection of oleaginous yeasts with high lipid productivity for practical biodiesel production Bioresour Technol 2014 153 230 235 24368271 10.1016/j.biortech.2013.11.086

[B26] Rossi M Buzzini P Cordisco L Amaretti A Sala M Raimondi S Ponzoni C Pagnoni UM Matteuzzi D Growth, lipid accumulation, and fatty acid composition in obligate psychrophilic, facultative psychrophilic, and mesophilic yeasts FEMS Microbiol Ecol 2009 69 363 372 10.1111/j.1574-6941.2009.00727.x 19624740

[B27] Samul D Leja K Grajek W Impurities of crude glycerol and their effect on metabolite production Ann Microbiol 2013 in press, doi:10.1007/s13213-013-0767-x 10.1007/s13213-013-0767-x PMC4119583 25100926

[B28] Rumbold K van Buijsen HJ Overkamp KM van Groenestijn JW Punt PJ van der Werf MJ Microbial production host selection for converting second-generation feedstocks into bioproducts Microb Cell Fact 2009 8 64 10.1186/1475-2859-8-64 19958560 PMC2795742

[B29] Rywińska A Rymowicz W Zarowska B Skrzypiński A Comparison of citric acid production from glycerol and glucose by different strains of *Yarrowia lipolytica* World J Microbiol Biotechnol 2010 26 1217 1224 10.1007/s11274-009-0291-0 24026926

[B30] Liu Y Koh CM Ji L Bioconversion of crude glycerol to glycolipids in *Ustilago maydis* Bioresour Technol 2011 102 3927 3933 10.1016/j.biortech.2010.11.115 21186122

[B31] Saenge C Cheirsilp B Suksaroge TT Bourtoom T Potential use of the oleaginous red yeast *Rhodotorula glutinis* for the bioconversion of crude glycerol from biodiesel plant to lipids and carotenoids Process Biochem 2011 46 210 218 10.1016/j.procbio.2010.08.009

[B32] Xu J Zhao X Wang W Du W Liu D Microbial conversion of biodiesel byproduct glycerol to triacylglycerols by oleaginous yeast *Rhodosporidium toruloides* and the individual effect of some impurities on lipid production Biochem Eng J 2012 65 30 36

[B33] Fakas S Papanikolaou S Batsos A Galiotou-Panayotou M Mallouchos A Aggelis G Evaluating renewable carbon sources as substrates for single cell oil production by *Cunninghamella echinulata* and *Mortierella isabellina* Biomass Bioeng 2009 33 573 580 10.1016/j.biombioe.2008.09.006

[B34] Beopoulos A Cescut J Haddouche R Uribelarrea JL Molina-Jouve C Nicaud JM *Yarrowia lipolytica* as a model for bio-oil production Prog Lipid Res 2009 48 375 387 10.1016/j.plipres.2009.08.005 19720081

[B35] Li Y Zhao ZK Bai F High-density cultivation of oleaginous yeast *Rhodosporidium toruloides* Y4 in fed-batch culture Enz Microb Technol 2007 41 312 317 10.1016/j.enzmictec.2007.02.008

[B36] Ashby RD Nunez A Solaiman DKY Foglia TA Sophorolipid biosynthesis from a biodiesel co-product stream J Am Oil Chem Soc 2005 82 625 630 10.1007/s11746-005-1120-3

[B37] Yang LB Zhan XB Zheng ZY Wu JR Gao MJ Lin CC A novel osmotic pressure control fed-batch fermentation strategy for improvement of erythritol production by *Yarrowia lipolytica* from glycerol Bioresour Technol 2014 151 120 127 24215768 10.1016/j.biortech.2013.10.031

[B38] Satyarthi JK Srinivas D Ratnasamy P Estimation of free fatty acid content in oils, fats, and biodiesel by 1H NMR spectroscopy Energy Fuels 2009 23 2273 2277 10.1021/ef801011v

[B39] Kohlwein SD Veenhuis M van der Klei IJ Lipid droplets and peroxisomes: key players in cellular lipid homeostasis or a matter of fat - store ‘em up or burn ’em down Genetics 2013 193 1 50 10.1534/genetics.112.143362 23275493 PMC3527239

[B40] Ma F Hanna MA Biodiesel production: a review Bioresour Technol 1999 70 1 15 10.1016/S0960-8524(99)00025-5

[B41] Khot M Kamat S Zinjarde S Pant A Chopade B Ravikumar A Single cell oil of oleaginous fungi from the tropical mangrove wetlands as a potential feedstock for biodiesel Microb Cell Fact 2012 11 71 10.1186/1475-2859-11-71 22646719 PMC3442963

[B42] Makri A Fakas S Aggelis G Metabolic activities of biotechnological interest in Yarrowia lipolytica grown on glycerol in repeated batch cultures Bioresour Technol 2010 101 2351 2358 10.1016/j.biortech.2009.11.024 19962884

[B43] Zhang W Bevins MA Plantz BA Smith LA Meagher MM Modeling *Pichia pastoris* growth on methanol and optimizing the production of a recombinant protein, the heavy-chain fragment C of botulinum neurotoxin, serotype A Biotechnol Bioeng 2000 70 1 8 10.1002/1097-0290(20001005)70:1<1::AID-BIT1>3.0.CO;2-Y 10940857

[B44] Trevelyan WE Forrest RS Harrison JS Determination of Yeast Carbohydrates with the Anthrone Reagent Nature 1952 170 626 627 10.1038/170626a0 13002392

[B45] Lapuerta M Rodríguez-Fernández J Armas O Correlation for the estimation of the density of fatty acid esters fuels and its implications. A proposed Biodiesel Cetane Index Chem Phys Lipids 2010 163 720 727 10.1016/j.chemphyslip.2010.06.004 20599853

[B46] Knothe G Steidley KR Kinematic viscosity of fatty acid methyl esters: prediction, calculated viscosity contribution of esters with unavailable data, and carbon–oxygen equivalents Fuel 2011 90 3217 3224 10.1016/j.fuel.2011.06.016

[B47] Tong D Hu C Jiang K Li Y Cetane number prediction of biodiesel from the composition of the fatty acid methyl esters J Am Oil Chem Soc 2011 88 415 423 10.1007/s11746-010-1672-0

[B48] Demirbas A Relationships derived from physical properties of vegetable oil and biodiesel fuels Fuel 2008 87 1743 1748 10.1016/j.fuel.2007.08.007

