# Supplementary material for: Getting lipids from glycerol: new perspectives on biotechnological exploitation of Candida freyschussii
Source: Microb Cell Fact. 2014 Jun 7;13:83. doi: 10.1186/1475-2859-13-83 (PMC4064286; doi:10.1186/1475-2859-13-83)
Supplement: Additional file 1: Table S1 — Calculation of biodiesel physicochemical properties from the composition of the fatty acids methylesters. [file 1475-2859-13-83-S1.pdf]

Calculation of biodiesel physicochemical properties from the composition of the fatty acids methylesters

The physicochemical properties of the mixtures of fatty acids methylesters were calculated using predictive equations (above) and database values of individual pure fatty acids methylesters (below). c_i is the relative composition in mass, the subscript (i) indicating each individual methylester.

Mixtures of fatty acid methylesters

Property	Equation	References
Density at 15°C, g/L	$\rho_{mix} = \sum_i (c_i \cdot \rho_i)$	41,45
Kinematic Viscosity at 40°C, mm^2/s	$v_{mix} = \sum_i (c_i \cdot v_i)$	41,46
Saponification Number	$SN_{mix} = \sum_i (c_i \cdot 560)/MW_i$	41
Iodine Number	$IV_{mix} = \sum_i (c_i \cdot D_i \cdot 254)/MW_i$	41
Cetane Number	$CN_{mix} = 1.068 \cdot \sum_i (c_i \cdot CN_i) - 6.747$	41,47
Higher Heating Value, MJ/Kg	$HHV_{mix} = 49.43 - (0.041 \cdot SN_{mix} + 0.015 \cdot IV_{mix})$	48
Flash Point, °C	$FP_{mix} = (HHV_{mix} - 32.12)/0.021 - 273.15$	48

Individual fatty acid methylesters

Property		C16	C16:1	C18	C18:1	C18:2	C18:3	References
Double bonds	D_i	0	1	0	1	2	3	
Molecular weight, g/mol	ρ_i	270.5	268.4	298.5	296.5	294.5	292.5	
Cetane Number	CN_i	74.3	51.0	75.6	56.5	38.2	22.7	47
Density at 15°C, g/L	ρ_i	864.2	882.4	867.6	877.5	893.2	901.8	45
Kinematic viscosity at 40°C, mm^2/s	v_i	4.4	3.7	5.9	4.5	3.7	3.1	46
